# Shelters and Their Use by Fishes on Fringing Coral Reefs

**DOI:** 10.1371/journal.pone.0038450

**Published:** 2012-06-20

**Authors:** Alexandre Ménard, Katrine Turgeon, Dominique G. Roche, Sandra A. Binning, Donald L. Kramer

**Affiliations:** 1 Department of Biology, McGill University, Montréal, Québec, Canada; 2 Department of Integrative Biology, University of Guelph, Guelph, Ontario, Canada; 3 Australian Research Council Centre of Excellence for Coral Reef Studies, Research School of Biology, The Australian National University, Canberra, Australian Capital Territory, Australia; Leibniz Center for Tropical Marine Ecology, Germany

## Abstract

Coral reef fish density and species richness are often higher at sites with more structural complexity. This association may be due to greater availability of shelters, but surprisingly little is known about the size and density of shelters and their use by coral reef fishes. We quantified shelter availability and use by fishes for the first time on a Caribbean coral reef by counting all holes and overhangs with a minimum entrance diameter ≥3 cm in 30 quadrats (25 m^2^) on two fringing reefs in Barbados. Shelter size was highly variable, ranging from 42 cm^3^ to over 4,000,000 cm^3^, with many more small than large shelters. On average, there were 3.8 shelters m^−2^, with a median volume of 1,200 cm^3^ and a total volume of 52,000 cm^3^m^−2^. The number of fish per occupied shelter ranged from 1 to 35 individual fishes belonging to 66 species, with a median of 1. The proportion of shelters occupied and the number of occupants increased strongly with shelter size. Shelter density and total volume increased with substrate complexity, and this relationship varied among reef zones. The density of shelter-using fish was much more strongly predicted by shelter density and median size than by substrate complexity and increased linearly with shelter density, indicating that shelter availability is a limiting resource for some coral reef fishes. The results demonstrate the importance of large shelters for fish density and support the hypothesis that structural complexity is associated with fish abundance, at least in part, due to its association with shelter availability. This information can help identify critical habitat for coral reef fishes, predict the effects of reductions in structural complexity of natural reefs and improve the design of artificial reefs.

## Introduction

In coral reef ecosystems, structural complexity is frequently associated with greater abundance and number of fish species [1]–[4]. One hypothesis for this association is that complex structures offer more shelters or refuges such as holes, caves, and crevices, which provide protection from predators, competitors, currents, and strong light as well as sites for reproduction and foraging [5]–[8]. Supporting evidence comes from a small number of observational studies demonstrating that measures of shelter availability such as density or total volume of holes on natural reefs predict abundance or species richness better than other measures of physical complexity such as rugosity [2], [9], [10]. In addition, there is support from experimental studies on a variety of small, artificially constructed reefs showing an increase in fish density with increasing shelter availability [7], [11]–[15]. Yet, despite the potential importance of shelters for fishes, information about their distribution and abundance is remarkably scarce for natural coral reefs and nonexistent in the Caribbean region (but see [16]). This gap in knowledge may be due in part to the challenges of defining shelters (e.g., [17]) and to the time and effort required to measure and count them [2], [10]. Among the studies that did measure and count shelters on reefs, very few presented the data and instead provided only correlations, qualitative indices or integrated measures from ordination analyses [2], [10], [18]–[21], preventing comparisons among studies. To the best of our knowledge, no studies have examined variables that influence shelter availability other than coral cover and most studies of shelter use by coral reef fishes have focused on one or a few related species (e.g., [17], [22]–[26]). No study has attempted to identify the whole assemblage of shelter-using fishes or to document the variables that influence which of the available shelters are used. Assessing the variables influencing shelter availability and occupation by fishes is important for identifying critical habitat for conservation, for understanding the ecological implications of reductions in reef complexity [27], and for improving the design of artificial reefs. This information is particularly critical for Caribbean reefs which, despite being among the best studied in the world, are also among the most threatened [28].

The goals of our study were (1) to assess the size distribution of two types of shelters (holes and overhangs) in a fringing reef system in Barbados, (2) to examine how shelter occupancy by fishes was related to shelter size and type, (3) to determine how spatial variation in shelter availability, as measured by shelter density, shelter size, and total shelter volume, was related to structural complexity, reef zone and water depth, (4) to determine how spatial variation in the proportion of shelters occupied and in the density of shelter-using fishes was related to shelter availability and to structural complexity, reef zone and water depth, (5) to examine evidence that shelters are a limiting resource as indicated by the relationship between shelter availability and fish density, and (6) to characterize the species richness and diversity of the shelter-using fish assemblage.

## Methods

### Ethic Statement

This study involved no capture or handling of fishes or corals and only brief disturbance of fishes when sampling shelter characteristics. The procedures were approved by the McGill University Animal Use Committee, Animal Use Protocol and Permit 5039 and conformed to all guidelines of the Canadian Council on Animal Care.

### Study Sites

**Figure 1:**
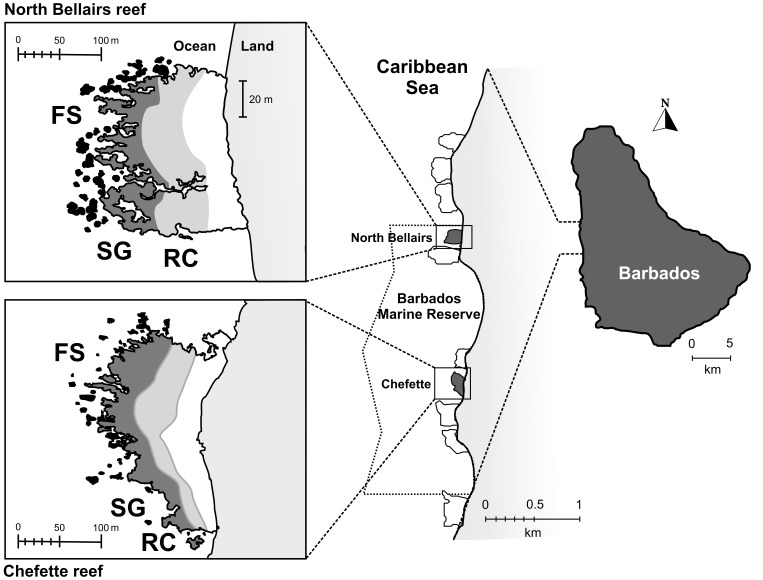
Schematic representation of the two study reefs on the west coast of Barbados, West Indies. The three reef zones examined are indicated as follows: reef crest (RC, light grey), spur and groove (SG, dark grey), and fragmented spurs (FS, black). The back reef (white), located inshore of the reef crest, was not sampled in this study. The Barbados Marine Reserve is indicated by the dotted polygon.

We sampled shelters in three zones of two fringing reefs on the west coast of Barbados, West Indies. Barbados has well-defined fringing coral reefs on its west (leeward) coast, which have become partially degraded since first described in the 1960s [29]–[31]. The reefs sampled were North Bellairs (13°11′33″ N, 59°38′30″ W) and Chefette (13°10′53″ N, 59°38′25″ W), both in the Barbados Marine Reserve (Figure 1). The data were collected using SCUBA in June – August 2006 between 08:30 hrs and 16:30 hrs on days when the visibility was at least 5 m.

We sampled only the reef crest, spur and groove and fragmented spur zones. The back reef zone (termed reef flat by Lewis [29]) was excluded because we wished to focus our efforts on areas with greater densities of shelters and fishes. Zones were identified by observations of the physical characteristics of the substrate prior to sampling and comparisons with distance from shore measurements of each zone from previous research in Barbados ([29], [31], [32]; Figure 1). The reef crest extends seaward, approximately 40 m from the edge of the back reef. Unlike offshore or exposed reefs, the reef crest on the leeward side of Barbados seldom experiences heavy wave action. The surface is exposed in places during extreme low tides but usually remains about 1 m below the surface. The substrate is composed mainly of dead coral rock with irregular surfaces and small pinnacles of coralline algae (predominantly *Porolithon*) coating the remains of coral skeletons. The crest as defined in this study combines parts of the reef crest and the coalesced spur zone described by Lewis [29] and Tomascik and Sander [31] and corresponds to the reef flat of Stearn et al. [32]. The spur and groove zone consists of a series of ridges projecting seaward and alternating with winding valleys of sand and rubble [29], [31], [32]. The spurs support substantial live coral cover and reach depths of 3–4 m at the seaward edge. We distinguished a fragmented spur zone immediately seaward from the end of the continuous spurs. Here, the continuous ridges disappear and are replaced by scattered patches of coral heads, often a single massive colony of *Montastrea*, *Siderastrea* or *Diploria* surrounded by sand. Although this habitat was considered part of the spur and groove zone by previous authors, we distinguished it because of its greater depth and patchy structure.

We used gridded aerial maps of the sites to delimit the study areas and select GPS coordinates of potential sampling locations. We chose fifteen quadrats on each reef by randomly selecting GPS coordinates from the maps. A marker was dropped from a boat above each selected position and a diver located the marker (representing the center of the quadrat) underwater to determine whether the site was suitable for sampling. Since our purpose was to focus on habitat dominated by hard substrate within each zone, we excluded quadrats for which the estimated sand cover exceeded 50%. In the very few instances when this occurred, we chose another quadrat by randomly selecting a new GPS coordinate. This randomized sampling did not produce a balanced design across reef zones; instead, it reflected the relative contribution of each zone to the habitat on the two reefs. Of the 30 quadrats selected, 17 were in the reef crest, 7 in the spur and groove zone and 6 in the fragmented spurs.

### Data Collection

When the center of a quadrat was selected, we determined its boundaries (5×5 m) with a measuring tape and marked the corners with flagging tape. Following a 10-min habituation period for fishes to resume normal activity, two divers began measuring shelters and recording their occupants. A shelter was defined as any enclosed or semi-enclosed space, including holes, crevices and spaces under overhanging structures and between branches of living coral. We only sampled shelters for which the smallest diameter of the entrance was at least 3 cm because of the time and effort necessary to sample the numerous very small shelters in our relatively large quadrats. For shelters with more than one entrance, we used the largest entrance for measures of location, size and depth. Shelters were classified into two types based on the amount of lateral protection offered: holes had walls on all but one side, whereas overhangs were spaces under projections without front or lateral walls. For each shelter, we recorded the XY coordinates of the shelter entrance within the quadrat (±5 cm) and the number and species of all fishes occupying it. A shelter was considered occupied if a fish was at least partially inside it when the sampling began. We then measured (±1 cm) the width and height of the entrance (holes) or the width and height of the covered space (overhangs) as well as the distance from the entrance of a hole or front of an overhang to the end of the shelter (length) using a graduated PVC tube. We estimated shelter volume using the formula for the area of an ellipse (0.7854 width×height) multiplied by the length of the shelter [2]. Fish that swam out during measurements were included in the count, but the occasional fish that entered a shelter during a measurement was not. If a fish swam between multiple shelters, we recorded all shelters used by the individual during the sampling period and randomly assigned the fish to a single shelter for the analyses. For each quadrat (25 m^2^), we combined holes and overhangs and calculated three measures of the amount of space available in shelters: the mean density of shelters (number m^−2^), the median volume of shelters (cm^3^), and the total volume of all shelters combined (cm^3^ m^−2^). We used the median volume because shelter volume was log-normally distributed. We calculated two measures of shelter use by fishes: the proportion of shelters occupied by one or more fishes and the density of shelter-using fishes (number m^−2^).

We recorded the mean water depth and structural complexity for each quadrat. Mean water depth was calculated from readings on a dive computer (Suunto Gekko Watch) at all intersections of a 1×1 m grid (36 measurements per quadrat). Structural complexity was estimated using a modification of rugosity measurements described by Luckhurst and Luckhurst [1]. A 5 m chain was laid along three length and three width positions (2.5 m apart) on the quadrat (six measurements in total), and the horizontal distance covered by the chain at each position was determined. Rugosity was calculated as the stretched length of the chain (500 cm) divided by the horizontal distance the chain covered when laid along the contour of the reef. Two divers required an average of 4.5 h (range 1.6–8.9 h) to complete the measurements on a quadrat, depending on the number of shelters present.

### Data Analysis

For quantitative descriptions of shelters and shelter-using fishes, we provide means where the data were approximately normally distributed and medians where the data were approximately log-normally distributed.

#### Variation in shelter volume

To test for differences in volume between holes and overhangs, we used a generalized linear mixed model (GLMM) (glmer function in lme4 package in Rv2.12.2 [33]) with a Gaussian structure of error terms. Quadrat, reef zone (crest vs. spur and groove vs. fragmented spur) and reef identity (North Bellairs vs. Chefette) were treated as random factors, and we controlled for spatial autocorrelation by blocking shelters (n = 2,863) by quadrat and nesting quadrats within reef zone and reef identity.

#### Occupancy and number of occupants in relation to shelter volume

We examined the relationships between shelter volume and (1) shelter occupancy and (2) the number of fish per occupied shelter, including the effect of shelter type. We used GLMMs with a binomial or a Gaussian error term structure, as appropriate. We controlled for spatial autocorrelation by blocking shelters (n = 2,863) or occupied shelters (n = 1,266) by quadrat and nesting quadrats within reef zone and reef identity.

#### Predictors of shelter availability

We used the Information Theoretic approach [34] to determine which set of physical variables best explained variation in shelter availability as measured by (1) shelter density, (2) median shelter volume and (3) total shelter volume, using quadrats as replicates (n = 30). We used GLMMs with three predictors: rugosity, reef zone, and reef identity. For these analyses, reef zone and identity were used as both fixed and random factors, where zone was nested within reef identity, to account for the spatial autocorrelation of quadrats. The fragmented spur zone was used as the treatment contrast for the factor reef zone, and North Bellairs was used as the treatment contrast for the factor reef identity. Because the previous analyses had revealed few significant differences between shelter types, we combined holes and overhangs for these analyses. Prior to each analysis, for model simplicity and parsimony and following recommendation from Burnham and Anderson [34], we reduced the number of candidate models in our analysis by excluding single terms and two-way interactions that had no apparent effect on the response variable as determined from graphical examination of all biologically meaningful two-way interactions. To select the best candidate models for each response variable, we used the Akaike Information Criterion modified for small sample sizes (AICc) and performed model averaging when the normalized Akaike weight values (*w_im_*) of the best models were <0.9 ([34], [35]; Text S1). We built distinct sets of models with water depth and reef zone to avoid problems of multicollinearity, but present only the results of models with reef zone which had consistently higher predictive power and support, based on AICc scores. We used the percent deviance explained to evaluate each model’s goodness-of-fit. We allowed a two-way interaction term between rugosity and reef zone. Rugosity, median shelter volume and total shelter volume were log_10_-transformed prior to the analyses. Rugosity and water depth were z-standardized (i.e. mean = 0, SD = 1) to remove non-essential collinearity between single predictors and interaction terms [36], to facilitate comparison among predictors by converting them to a similar scale, and to make single terms more interpretable in the presence of an interaction [37].

#### Predictors of occupancy and density of shelter-using fishes

We performed similar analyses as above (*Predictors of shelter availability*) to test which physical characteristics of reefs and shelters best explained variation in (1) the proportion of shelters occupied and (2) the density of shelter-using fishes, using quadrats as replicates (n = 30). Six predictors were included in each analysis: shelter density, median shelter volume, total shelter volume, rugosity, reef identity, and reef zone or water depth. Two-way interactions were allowed between reef zone and shelter density, median shelter volume and rugosity, total shelter volume and rugosity, water depth and rugosity, shelter density and median shelter volume, and shelter density and total shelter volume. We only present models with reef zone because zone consistently explained a higher percentage of the total deviance and had better support than water depth. Rugosity, median shelter volume, and total shelter volume were log_10_-transformed and z-standardized prior to the analyses. Shelter density was also z-standardized.

#### Shelters as a limiting resource for fishes

We examined whether shelters are a limiting resource for fishes on the reef by determining the shape of the relationships (linear vs. asymptotic) between fish density and (1) shelter density, (2) median shelter volume, and (3) total shelter volume. A linear relationship between population density and resource availability would be expected for a limiting resource whereas population density should have no relationship or an asymptotic relationship with a non-limiting resource. We estimated the parameter value *b* for the linear relationships (y = b*x) and the parameters *b* and *d* for the asymptotic relationships (y = (b*x/[1+(*b*/d*x)]) using a maximum likelihood approach (function mle2 in R, bbmle package v1.0.0; [38]). We chose a normal distribution (dnorm) to model our data and assessed the likelihood of each model with AICc scores and normalized Akaike weights (*w_im_*).

#### Species richness

Species richness estimates typically increase with increasing sample size before reaching an asymptote [39]. To test whether the number of shelters sampled in each quadrat was sufficient to assess species richness at this scale, we produced a rarefaction curve [39], which related the mean and standard deviation of the expected number of species observed to the number of occupied shelters sampled in all 30 quadrats combined. The curve was calculated based on random permutations of the entire dataset using the specaccum function in R (vegan package; [40]).

## Results

### Variation in Shelter Volume

**Figure 2:**
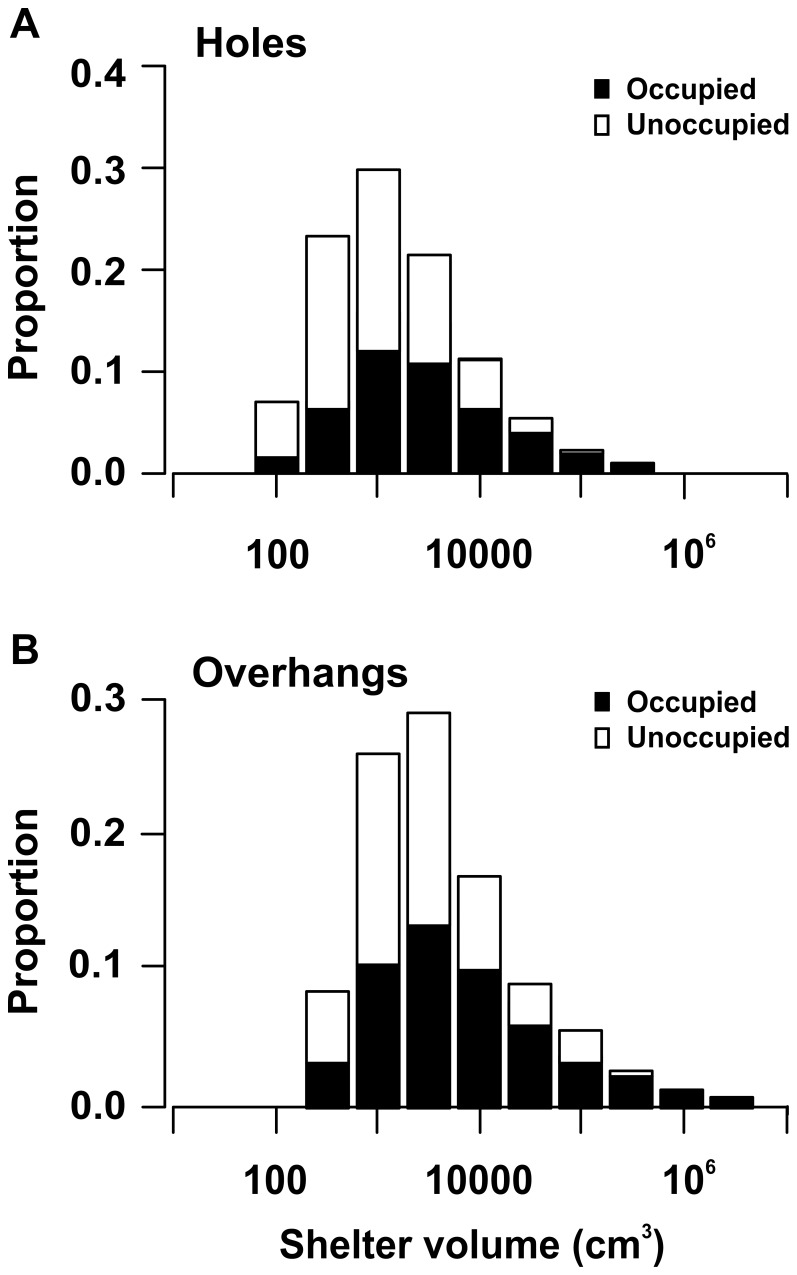
Frequency of holes and overhangs in relation to shelter volume. The proportional distribution of (A) holes (n = 2,134) and (B) overhangs (n = 729) in relation to shelter volume. Occupied shelters are shown as black bars and unoccupied shelters as white bars. Each bin has a width of 0.5 log_10_ cm^3^. Bins with values smaller than 100 cm^3^ are not shown because proportions were less than 0.001.

Individual shelter volumes varied by nearly 5 orders of magnitude, with the smallest shelter measuring 42 cm^3^ and the largest over 4,000,000 cm^3^ (4 m^3^). There were nearly three times as many holes (n = 2,134) as overhangs (n = 729). The volumes of both holes and overhangs were approximately log-normally distributed, indicating that there were many more small than large shelters (Figure 2). Although the size of holes and overhangs overlapped extensively, holes were smaller (median = 898 cm^3^), on average, than overhangs (median = 2,205 cm^3^; t-value = 187.52, estimate ± SE = 3.055±0.441, 95% CI = 0.377 to 0.504). Because some previous studies measured only shelter diameter, we examined the relationships among the measures of shelter size. For both holes and overhangs, width, height, and length of shelters were correlated with each other (Pearson correlations, r = 0.586–0.709) and with shelter volume (r = 0.840–0.907). Shelter volume was related to the largest diameter (width or height) by the relationship: log_10_ volume (cm^3^) = 0.553+2.252 log_10_ diameter (cm) (r^2^ = 0.847, 95% CI for intercept = 0.512 to 0.594, 95% CI for slope = 2.217 to 2.287).

Median shelter volumes per quadrat were log-normally distributed and varied 33-fold, ranging from 484 to 16,136 cm^3^ (median = 1,211 cm^3^). Mean shelter volumes were also log-normally distributed, but higher and more variable, with a 57-fold range from 1,125 to 64,517 cm^3^ (median = 12,492 cm^3^). Quadrat 5 on the crest on Chefette Reef had a much higher median shelter volume than the other 29 quadrats. Without this quadrat, the variation in median shelter volume was reduced to 9-fold, ranging from 484 to 4,432 cm^3^ (median = 1,188 cm^3^). Total shelter volume per quadrat was also log-normally distributed and varied 127-fold, ranging from 2,436 to 309,679 cm^3^⋅m^−2^ (median = 51,567 cm^3^⋅m^−2^).

### Occupancy and Number of Occupants in Relation to Shelter Volume

Of the 2,863 shelters examined, 44.2% were occupied by at least one fish, including 900 holes (42.2%) and 366 overhangs (50.2%). The proportion of occupied shelters increased from about 0.22 to 1.00 as shelter volume increased from about 100 cm^3^ to about 100,000 cm^3^ (Figure 2). Shelter volume (n = 2,863) explained 16.0% of the total deviance in occupancy (estimate ± SE = 0.637±0.046, z-value = 14.00, 95% CI = 0.691 to 0.905). There were no differences in shelter occupancy between holes and overhangs when controlling for shelter volume (estimate ± SE = 0.002±0.096, z-value = 0.026, 95% CI =  −0.185 to 0.190). The relationship between occupancy and shelter volume also did not differ between holes and overhangs, although there was a trend toward a faster increase in occupancy with increasing volume for holes than for overhangs (estimate ± SE =  −0.187±0.100, z-value =  −1.867, 95% CI =  −0.384 to 0.009).

**Figure 3:**
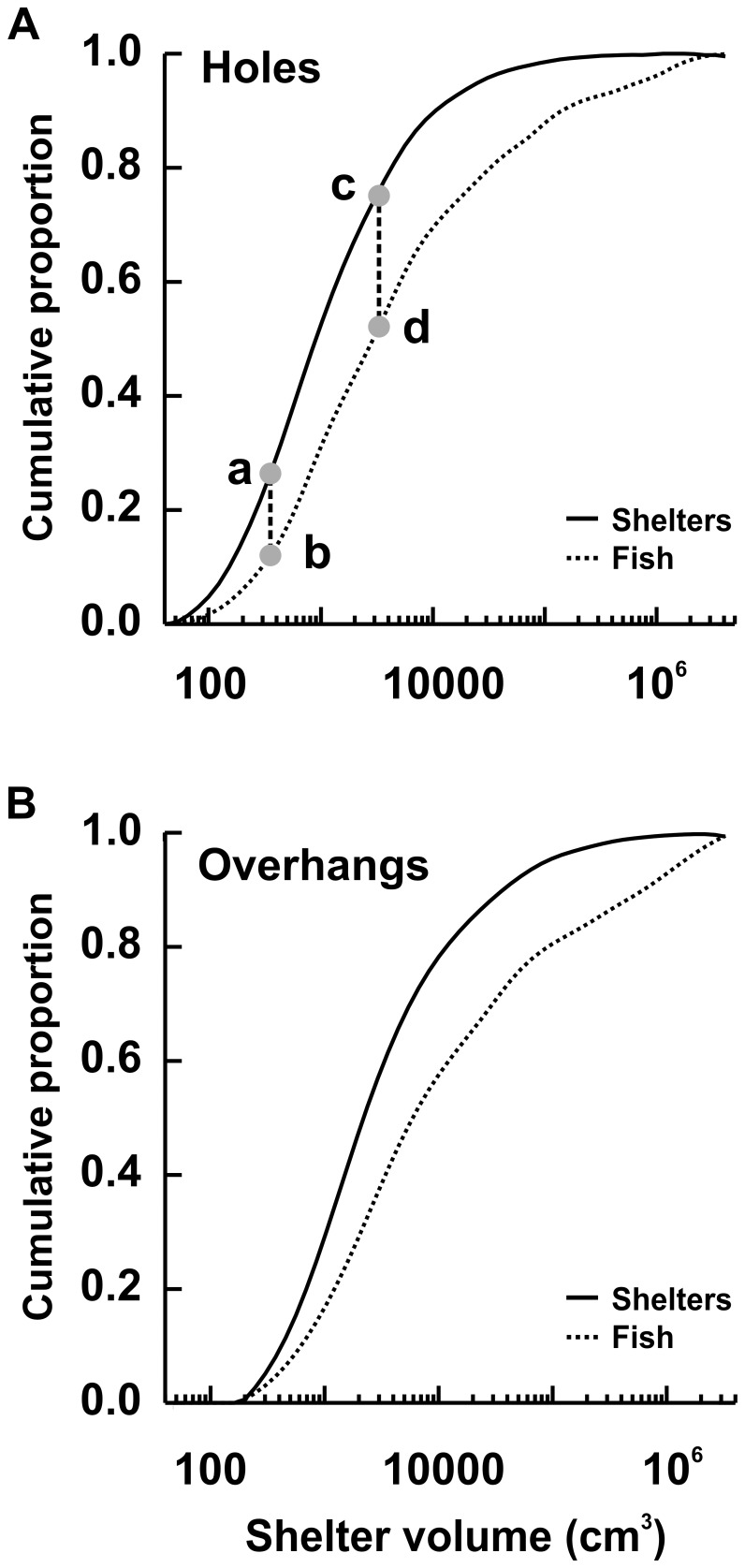
Cumulative proportion of shelters and shelter-using fishes in relation to shelter volume. The cumulative proportion of shelters with minimum entrance diameter >3 cm (solid lines) and the cumulative proportion of shelter-occupying fishes (dotted lines) in relation to log_10_ shelter volume for (A) holes (N = 2,134) and (B) overhangs (N = 729). The grey dot (labelled “a”) indicates the smallest 25% of shelters and corresponds to only 12% of the shelter-dwelling fishes in holes (labelled “b”). The largest 25% of shelters (above point “c”) correspond to approximately 50% of the shelter-dwelling fishes in holes (above point “d”).

The number of fishes per occupied shelter was log-normally distributed and ranged from 1 to 35, with a median of 1 fish per occupied shelter. Larger shelters were occupied by more fish. For example, the largest holes (upper quartile) contained approximately 50% of fishes found in holes, whereas the smallest holes (lower quartile) contained only 12% of these fishes (Figure 3A). A similar trend was observed for overhangs (Figure 3B). Shelter volume (n = 1,266) explained 7.8% of the total deviance in the number of fishes per occupied shelter (estimate ± SE = 0.2499±0.017, t-value = 14.58, 95% CI = 0.216 to 0.283). There were no differences in the number of fishes occupying holes versus overhangs when controlling for shelter volume (estimate ± SE = −0.102±0.041, t-value = −2.500, 95% CI = −0.182 to −0.022). The relationship between the number of fishes per occupied shelter and shelter volume also did not differ between holes and overhangs.

### Predictors of Shelter Availability among Quadrats

Mean shelter density per quadrat varied more than 13-fold, ranging between 0.6 and 8.2 shelters m^−2^ (mean = 3.8 shelters m^−2^) across reefs and reef zones. Shelter density was negatively correlated with median shelter volume (r = −0.228) but positively correlated with total shelter volume per quadrat (r = 0.498). Median and total shelter volumes were uncorrelated (r = 0.010).

**Figure 4:**
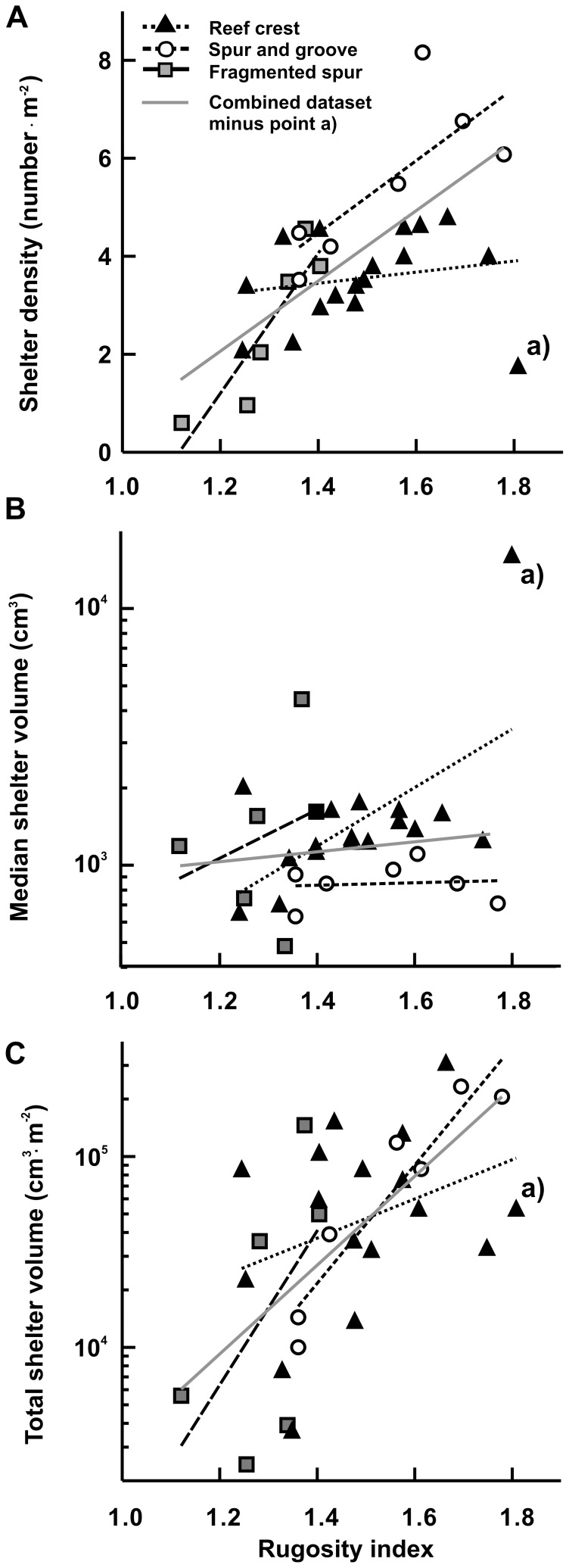
Shelter availability variables in relation to structural complexity. The relationship between A) shelter density (number m^−2^), B) median shelter volume (cm^3^), C) total shelter volume (cm^3^ m^−2^) and mean rugosity index across the 30 quadrats sampled. Lines represent the best fit linear regressions for each zone considered separately (reef crest – dotted, spur and groove – short dashes, fragmented spurs – long dashes). The solid gray line represents the best fit linear regression for the entire dataset, excluding the data point recorded at the highest value of rugosity (Quadrat 5, point a).

Two models had support in explaining shelter density, based on AICc scores. The best model, which included the predictors reef zone, rugosity and their interaction, was highly supported (*w_im_* = 0.80) and explained 76.9% of the total deviance in shelter density. The second best model (*w_im_* = 0.20) included reef identity, reef zone, rugosity, the interaction between reef zone and rugosity and explained 77.7% of the total deviance. Based on predictor estimates, there was strong support for an increase in shelter density with increasing rugosity (estimate ± SE: 2.443±0.396, t-value = 6.171, 95% CI = 1.667 to 3.219, Figure 4A). However, shelter density increased more slowly with increasing rugosity in the reef crest (estimate ± SE: −2.3642±0.420, t-value = −5.624, 95% CI = −3.188 to −1.540) and in the spur and groove zone (estimate ± SE: −1.990±0.454, t-value = −4.379, 95% CI = −2.880 to −1.099) than in the fragmented spur zone (Figure 4A). Shelter density also varied with reef zone and reef identity. Differences among reef zones were due to lower shelter density in the fragmented spur zone than in the reef crest and the spur and groove zones (Figure 4A). The 95% CI of the estimate for the effect of the reef crest versus the fragmented spur zone did not overlap zero (estimate ± SE: −1.443±0. 464, t-value = −3.113, 95% CI =  −2.251 to −0.534), whereas the estimate of the effect of the spur and groove versus the fragmented spur zone did (estimate ± SE: −0.034±0.817, t-value = −1.626, 95% CI = −1.786 to 0.166). There was a trend toward higher shelter density on North Bellairs Reef than on Chefette Reef, but the 95% CI of the estimate for the effect of reef identity overlapped zero (estimate ± SE: 0.1934±0. 222, t-value 0.871, 95% CI = −0.242 to 0.628). Repeating the analysis without Quadrat 5 (very high rugosity but low shelter density; Figure 4A, point a) did not change the results.

Much like shelter density, total shelter volume per quadrat increased with rugosity (estimate ± SE: 0.597±0.152, t-value  = 3.924, 95% CI  = 0.298 to 0.893). The model that included only rugosity had extremely high support based on AICc scores (*w_i_*  = 0.93) and explained 35.5% of the deviance in total shelter volume (Figure 4C). In contrast, the relationships between median shelter volume per quadrat and physical predictors were less clear. There was evidence for an interaction between zone and rugosity, whereby median shelter volume increased more slowly with increasing rugosity in the spur and groove zone than in the fragmented spur and the reef crest zones. Overall, there was strong support for an increase in median shelter volume with increasing rugosity as well as evidence for an effect of reef zone - the median shelter volume was lower in the spur and groove than in the reef crest and fragmented spur zones (Table S1A). However, these trends were affected by the high median shelter volume and high rugosity of Quadrat 5 (Figure 4B). When we repeated the analysis without Quadrat 5, rugosity had very little influence on median shelter volume, and the 95% CI of all predictor estimates overlapped zero (Table S1B).

Predictors of shelter occupancy and density of shelter-using fishes among quadrats.

The proportion of shelters occupied varied more than 3-fold among quadrats (n = 30), ranging from 0.22 to 0.72 (median  = 0.40). The mean density of shelter-using fishes at the quadrat scale varied 23-fold, ranging from 0.4 to 9.2 fish m^−2^ (median  = 2.3 fish m^−2^).

Predictors of the proportion of shelters occupied in quadrats (n = 30) were ambiguous because the results were affected by the extreme shelter volumes in Quadrat 5. When the analysis included all quadrats, shelter occupancy appeared to be affected by median shelter volume and the interaction between median shelter volume and shelter density. Occupancy increased with median shelter volume and was higher when shelter density was also high (Table S2A, Figure S1A). However, after excluding Quadrat 5 from the analysis, total shelter volume was the strongest predictor, although none of the predictors had strong support because the 95% CI of all estimates overlapped zero (Table S2B).

**Figure 5:**
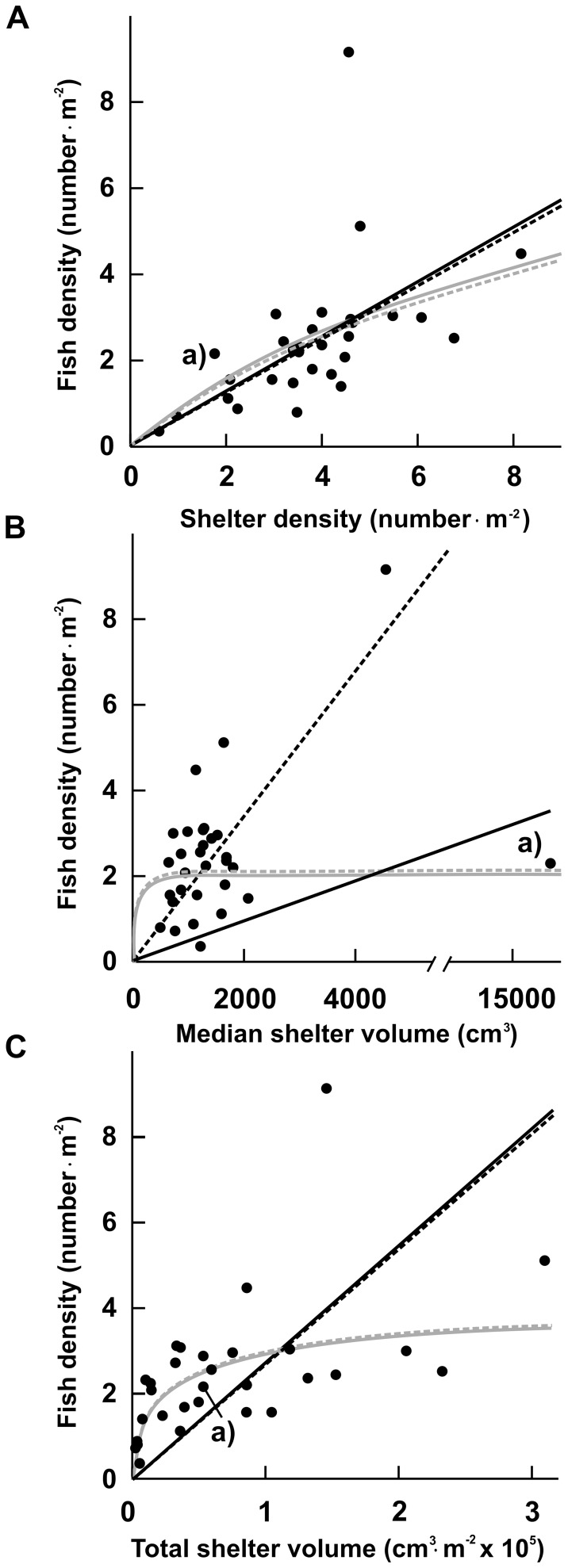
Relationship between fish density and shelter availability characteristics. The relationship between fish density (number m^−2^) and A) shelter density (number m^−2^), B) median shelter volume (cm^3^) and C) total shelter volume (cm^3^⋅m^−2^) for the 30 quadrats sampled. The solid black line represents the best fit linear regression to the entire dataset, whereas the dashed black line represents the best fit linear regression without Quadrat 5 (point a). The solid grey line represents the best fit asymptotic curve to the entire dataset, whereas the dashed grey line represents the best fit asymptotic curve without Quadrat 5 (point a).

Three models had support in explaining variation in fish density among quadrats (n = 30) based on AICc scores, explaining 74.4% to 83.9% of the total deviance (Table 1). Unlike the analysis for shelter occupancy, this analysis was not strongly affected by the extreme shelter volumes recorded in Quadrat 5. Median shelter volume, shelter density and the interaction between median shelter volume and shelter density were the most influential predictors; they were present in all models included in the best subset and the 95% CI of their estimates after model averaging did not overlap zero. Fish density increased with increasing median shelter volume and shelter density and was higher when both predictors were high. The univariate relationships between fish density and the three measures of shelter availability are shown in Figure 5, and the interaction is graphed in Figure S1B. Fish density was greater in the reef crest and spur and groove zones than in the fragmented spur zone; it was also higher on North Bellairs Reef than on Chefette Reef. The 95% CI for the estimates of reef zone and reef identity did not overlap zero. The model relating fish density to shelter density, median shelter volume and their interaction had over 1000 times more support than the models based on rugosity combined with any one of the three individual measures of shelter volume. It also had over 100 times more support than the model that included shelter density and median shelter volume, without their interaction.

**Table 1:** Fish density.

Predictors	Model Rank			β	SE	95% CI	wip
	1	2	3				
*Constant*	•	•	•	−0.750	−0.938	−0.220 to 1.189	1.00
Median shelter volume	•	•	•	0.886	0.132	0.628 to 1.144	1.00
Shelter density	•	•	•	0.631	0.116	0.404 to 0.857	1.00
Zone RC vs. FS	•	•		0.767	0.214	0.348 to 1.185	0.93
Zone SG vs. FS	•	•		1.105	0.356	0.406 to 1.803	0.93
Reefs NB vs. CH	•			0.296	0.150	0.003 to 0.589	0.64
Shelter volume * shelter density	•	•	•	0.545	0.118	0.313 to 0.779	1.00
No. of parameters (K)	8	7	5				
AICc	38.371	49.992	52.680				
ΔAICc	0.000	1.621	4.309				
w*_im_*	0.641	0.285	0.074				
Deviance explained	83.9	79.7	74.4				

Predictors and interaction terms included in the three best models explaining variation in fish density in thirty 25 m^2^ quadrats located in three reef zones (RC = reef crest, SG = spur and groove, FS = fragmented spurs) and two reefs (NB = North Bellairs, CH = Chefette). Variables included in each model are denoted with “•”. Predictors for which the 95% confidence interval (CI) did not overlap zero are indicated in bold font. The number of parameters (K) used in each model, the AICc, the ΔAICc (AIC of model*_i_*−AIC of best model), the w*_im_* (normalized Akaike weights for each candidate model) and the deviance explained are shown at the bottom of the table. Model averaged estimates of parameters (β), unconditional standard errors (SE), 95% CI and the normalized Akaike weight for each predictor (w*_ip_*) are also shown. All models include a constant.

### Shelters as a Limiting Resource for Fishes

Fish density increased linearly with increasing shelter density (Figure 5A). Based on maximum likelihood estimation, the linear relationship between fish density and shelter density with a slope of 0.62 (ΔAICc = 0.0, *w_im_* = 0.686, total deviance explained = 26.3%) was 2.2 times more likely than the asymptotic relationship (ΔAICc = 1.6, *w_im_* = 0.314). Fish density also increased linearly with increasing median shelter volume (Figure 5B). The linear relationship with a slope of 0.01 had extremely high support (ΔAICc = 0.0, *w_im_* = 0.999) compared to the asymptotic relationship (ΔAICc = 23.9, *w_im_* = <0.001), but explained only 2.24% of the total deviance in fish density. After excluding Quadrat 5, the linear relationship had a slope of 0.04 and explained 52.9% of the total deviance. In contrast to shelter density and median shelter size, fish density increased non-linearly with increasing total shelter volume per quadrat. The asymptotic relationship had extremely high support (ΔAICc = 0.0, *w_im_* = 0.999) compared to the linear relationship (ΔAICc = 14.2, *w_im_* = <0.001). Fish density increased rapidly with increasing total shelter volume, reaching a density of approximately 3 fish m^−2^ at an approximate total shelter volume of 100,000 cm^3^ m^−2^ (Figure 5C). The asymptote for this relationship was approximately 3.9 fish m^−2^.

### Species Richness

In total, we recorded 1,845 shelter-using fishes belonging to 66 species (Table S3). The rarefaction curve (Figure S2) indicated that the number of species recorded rose steeply with the number of shelters sampled. On average, detecting 50% of the total number of species recorded in this study required sampling 140 occupied shelters. These numbers suggest that 5×5 m quadrats containing 4–82 occupied shelters (mean = 42.2) were too small a sampling area to provide a reliable estimate of species richness in the system. However, the large number of occupied shelters sampled in this study (n = 1,266) is sufficient to suggest that 66 species is a reliable estimate of species richness in the system.

## Discussion

### Shelter Sizes

Our study provides the first published data on the size distribution of shelters on a Caribbean reef, the first detailed documentation of the individual and total volumes of shelters on a reef, and the first comparison between the abundance and sizes of holes and overhangs. Larger shelters were much less abundant than smaller shelters. Frequency consistently decreased as shelter size increased for holes above 10,000 cm^3^ and for overhangs above about 32,000 cm^3^. Similar patterns were apparent in studies on natural reefs [9], coral heads [16], and small experimental reefs created with living coral [7]. Because we did not measure shelters <3 cm in minimum entrance diameter, we likely underestimated the frequency of shelters with very small volumes. Therefore, the decreasing frequency of shelters smaller than 10,000 cm^3^ does not provide evidence for a lower abundance of smaller shelters.

Shelter size and abundance differed between holes and overhangs. Holes were more numerous but smaller, on average, than overhangs. The correlations among the linear measurements and between the linear measures and volumetric estimate suggest that measuring only diameter may provide an approximate estimate of shelter volumes. This may be sufficient in many cases, especially since the volumetric calculation is only an approximation based on idealized geometry and not able to account for curving passages that penetrate deeper into the reef [41]. However, different types of shelters may differ in their relationship between entrance diameter and volume. Indeed, additional ecological insights might be gained by further refining the classification of shelter types, for example, by distinguishing shelters among the branches of living coral colonies from cavities of different origin within the reef structure [41].

Surprisingly few studies have quantified the size and availability of shelters on natural coral reefs, and even fewer have presented data allowing comparison among shelter types, reef types or geographical regions. Studies that included all potential shelters on reefs have been carried out on the Great Barrier Reef [18]–[21], the Red Sea [9], Hawaii [2], Moorea [42], and the Seychelles [10]. The only Caribbean observations have come from Nemeth’s [16] studies of how shelters in isolated heads of boulder coral *Montastrea annularis* and *Porites porites* rubble influence survival of newly settled damselfish *Stegastes partitus* in the U.S. Virgin Islands and Forrester and Steele’s [17] estimation of the abundance of crevices at the sand-reef interface suitable as refuges for the bridled goby *Coryphopterus glaucofraenum.* Of the studies that included all potential shelters, only two presented detailed size and density information [9], [16]. One study aggregated the data into a subjective ordinal scale comprising both shelter density and diversity [21], and the others presented the data only synthesized by means of a Principal Components Analysis [2], [10], [20], [42].

### Shelter Occupancy

Larger shelters were more likely to be occupied and, if occupied, to contain a larger number of fishes. The pattern did not differ between holes and overhangs when the difference in size between the two shelter types was taken into account. Holes in the two smallest size classes (<1,000 cm^3^) were occupied less than one third of the time, whereas holes in the two largest size classes (>100,000 cm^3^) were always occupied. Only 12% of shelter-using fishes were found in the smallest 25% of holes whereas 50% of fishes were found in the largest 25% of holes. While shelter size obviously limits the maximum number of occupying fish, this constraint seems unlikely to explain the observed pattern because many of the smaller shelters were large enough to be used by additional individuals. Although the effect of shelter volume was large, it only explained 8–16% of the deviance in occupancy and number of occupants, indicating the importance of other factors. Such factors might include aspects of shape, position, location on the reef and whether the shelter occurred in the reef matrix or in live coral. All these variables have been indicated as important in other studies [22], [23], [25].

We are not aware of any other studies that provide comparable data on shelter occupancy by fishes. Studies of occupation of artificial shelters by spiny lobsters *Panulirus argus* [43] and an assemblage of smaller invertebrates [44] have also found that larger shelters were generally occupied by more individuals. However, our results contrast with suggestions from previous studies emphasizing the value of a close match between shelter size and fish size [2], [12], [22]. Several possibilities may explain the higher rate of occupancy and higher number of fishes observed in larger shelters. Obviously, small shelters may physically exclude large individuals. Additionally, large shelters are rarer than small shelters and may therefore be heavily used by fish species or size classes that require large shelters. Larger shelters may also facilitate the formation of aggregations that provide antipredator defenses or other benefits as has been shown in spiny lobsters [43]. In addition, larger shelters may provide a greater range of microhabitats and may be harder to defend by territorial species that actively exclude other individuals. Nevertheless, more detailed studies are required to understand how shelter size and fish size are related for different taxa and different contexts.

### Predictors of Shelter Availability

Our study shows a considerably lower density of shelters than the few previous reports and is the first to examine predictors of spatial variation in shelter availability. On fringing reefs in Barbados, shelter density averaged 3.8 shelters m^−2^, median shelter volume about 1,200 cm^3^ and median total volume about 51,500 cm^3^. Only two studies provide data with which we can compare our measures, and both recorded only shelter density as a measure of shelter availability. Roberts and Ormond [9] recorded a much higher density of shelters, about 120 shelters m^−2^ (estimated from their Figure 7) on fringing reefs in the Red Sea. Even after excluding their 1–5 cm size class to make the data more comparable to our 3 cm threshold, there were still about 20 holes m^−2^. Because Roberts and Ormond [9] counted spaces between coral branches as shelters (C. Roberts, personal communication), the difference may be related to richer coral cover at their sites or to a higher proportion of solid substratum. Nemeth [16] reported that isolated *Montastrea* coral heads in the Virgin Islands averaged about 14 shelters m^−2^, but this probably included some shelters smaller than the size threshold we used.

Shelter availability showed important spatial variation among quadrats, with more than a 10-fold range for density and more than a 100-fold range for total volume. It appears that some quadrats had many small shelters whereas others had fewer but larger shelters, resulting in the weak negative correlation between shelter density and median size. However, density appeared to be more important than median size as an influence on the total volume of shelters because density and total volume were positively correlated whereas median size and total volume were not. Shelter density and total volume were clearly associated with rugosity, indicating that structural complexity reflects, in part, the presence of shelters. It is not clear whether median shelter size was also associated with rugosity because the positive association depended on a single data point. In the fragmented spur zone, shelter density was lower than in the other two zones, but increased more rapidly with increasing rugosity, indicating that rugosity cannot be taken as an absolute proxy for shelter density but needs to be related to zone. Variation in the relationship between rugosity and shelters could be a result of differences between zones in the amount of vertical relief and live coral. On the reefs examined, the reef crest included large eroded coral heads with high vertical relief whereas the fragmented spurs had low relief but more live coral and more shelter-rich interfaces between the reef and the sandy substratum. Recent studies indicate that rugosity varies with the type of coral cover and that changes in rugosity vary among processes that affect coral cover [45], [46]. To clarify the relationships between shelter availability and structural complexity, it may be useful to differentiate shelters located within the reef matrix from shelters formed among branches of living coral on the reef surface and to increase our understanding of the processes that create and destroy shelters. It appears that little is known about such processes, especially those occurring within the reef matrix (but see [41], [47]). We are aware of only one previous study that has attempted to examine the relationships between structural complexity and shelter availability. For the crevices at the reef-sand interface used by bridled gobies, Forrester and Steele [17] found that shelter density was associated with the proportion of solid substrate but only weakly with live coral cover and not at all with rugosity.

### Predictors of Shelter Occupancy and Fish Density

Our data suggest that the density of shelter-using fishes was directly related to shelter availability rather than to some other variable associated with structural complexity. Variation in the density of shelter-using fishes was quite well explained by a strong association with the density of shelters, their median size, and the interaction between density and median size, as well as some effect of reef zone and identity. Although rugosity was associated with shelter density, rugosity did not appear in the selected models and was less successful than shelter availability in predicting the density of shelter-using fishes. On the other hand, our models did not reveal robust predictors of occupancy, with the clearest pattern due to the effect of one extreme value. While many studies have found an association between rugosity and coral reef fish density [1], [4], [48], only a few have compared the predictive power of shelters with that of rugosity. Roberts and Ormond [9] found that a surface index similar in concept to rugosity but estimated from photographs had little predictive power for explaining the density of fishes at several sites and depths in the Red Sea; however, a multiple regression based on three size classes of holes explained much of the variance. Friedlander and Parrish [2] reported that fish density in Hawaii was much more strongly associated with the total volume of holes than with either rugosity or alternative measures of shelter availability. Wilson et al. [10] also found that fish density was more closely associated with principal components related to the density of holes than with rugosity on Seychelles reefs. Thus, our study confirms for Caribbean reefs and for the fish actually observed in shelters the positive associations between shelter availability and fish density that have been identified in several other regions. However, generalizations concerning which measures of shelter availability best predict fish density are not yet possible.

In addition to shelter characteristics, there was evidence of an effect of reef zone on the density of shelter-using fish. While spatial variation in fish density is not surprising, and reef zones and water depth are well known to influence reef fish abundance [49], our study shows that these spatial differences are not explained by differences in shelter availability and rugosity alone. Lower densities of fish in the fragmented spur zone may have been related to greater depth, lower vertical relief or the smaller proportion of continuous reef in this zone as well as to differences in shelter type. In addition, other variables that affect overall fish abundance such as the amount of live coral [50] may influence the density of shelter-using fishes.

### Shelters as a Limiting Resource for Fishes

The linear increase in the density of shelter-using fishes with increasing shelter density supports the hypothesis that shelters are a limiting resource for fishes on coral reefs [12], [13], [51]. Changes in the availability of a limiting resource are expected to have a linear effect on population size [52]. An asymptotic relationship would have indicated a reduction in the average number of fishes per shelter with increasing shelter density in quadrats, providing evidence that other resources or processes limited fish abundance [53]. The considerable number of unoccupied shelters is not evidence for a lack of limitation because some of the shelters may have been too small or unsuitable in other ways. Furthermore, territorial species such as some pomacentrids and holocentrids may defend multiple shelters, preventing some from being used [25], [54].

Previous studies have shown a positive association between shelter availability and coral reef fish density [2], [9], [10], but the shape of the relationship was not examined. Using observations plus experimental removal of shelters and increases in fish density, Robertson and Sheldon [22] did not find evidence for limitations in the availability of nocturnal shelters in the diurnal bluehead wrasse *Thalassoma bifasciatum*. On the other hand, studies of shelter addition [26], [55], density-dependent mortality in relation to shelter density [17], [56], and small, experimental artificial reefs [7], [11]–[14] have provided evidence that population density does increase with greater shelter availability in coral reef fishes. Thus, our study adds support for the hypothesis that shelters are sometimes limiting by applying it to the assemblage of shelter-using fishes and to spatial variation in fish density within larger, natural reefs.

### Species Richness

Most previous research on shelter use by coral reef fishes have either included all fish of broad taxonomic groupings without documenting whether or not they used shelters or have focused on one or a few related species. This study appears to be the first to survey shelters systematically and to record the associated fish assemblage. Of the 66 species of fish identified in shelters, the most commonly found were pomacentrids, especially the genus *Stegastes*. Pomacentrids are diurnal species that use shelters as refuges from predators during the day [57] and night [22] as well as for nest sites [58]. The abundant diurnal acanthurids, labrids and scarids found on Barbados fringing reefs [59] were never or very rarely observed in shelters. It is important to note that by sampling only during the daytime, our sample may have underrepresented shelter use by species that use shelters primarily at night [22], [60]. Other than pomacentrids, the majority of the fishes in shelters were apogonids, haemulids, holocentrids and serranids, taxa that are mostly nocturnal or crepuscular [25], [60]–[62]. Pempherids were rare in this sample but do occur in large aggregations in a few locations on these reefs. Because of the shelter size criterion we used, we did not record species associated with much smaller holes such as the chaenopsids [23]. Some taxa such as muraenids that spend much time within shelters will be underrepresented because they are often not visible to observers [63], probably because they spend time in deeper, narrow, or curving holes.

### Conclusions

Quantifying the size, number and use of shelters on two fringing reefs in Barbados has highlighted the importance of this component of habitat structure for the reef fish community. However, a lack of standardization in sampling methods, variables and the definition of a shelter on coral reefs make comparisons among studies difficult. We found that the rare, large shelters used by aggregations of several species have a disproportionate effect on fish densities and may be a valuable characteristic to assist in the selection of sites for conservation. This is even more important given the heavy impact and rapid changes occurring on coral reefs, particularly in the Caribbean region. The loss of structural complexity is a clear trend in the Caribbean, and possibly other regions [27]. If this loss reduces shelter availability, it may have profound effects on fish assemblages. However, our ability to predict such effects is limited because we know little about the processes responsible for the formation and loss of shelters, especially the larger caves, holes and crevices within the reef matrix. More detailed studies at the community level are also needed to help determine species preferences and their use of these important and limiting resources. We envisage considerable potential benefits from using artificial reefs to experimentally test the role of shelters of various sizes in the recovery of fish assemblages on damaged reefs.

## Supporting Information

Figure S1 **Shelter occupancy and fish density in relation to shelter density and median shelter volume.** Three-dimensional plots showing A) the proportion of shelters occupied and B) fish density as a function of shelter density (y-axis) and median shelter volume (x-axis) for the 30 quadrats sampled. Black dots represent individual quadrats and the relationships shown by the colored grid were extracted from a general linear model. Median shelter volume was log_10_ transformed and all variables were z-standardized. Point a) represents the extreme value of median shelter volume in Quadrat 5, which is discussed in the text.
(TIF)

Figure S2 **Rarefaction curve in shelter-using fish.** Shelter-based rarefaction curve (solid line) ± standard deviation (shaded area) relating the expected number of species observed to the number of occupied shelters sampled across all 30 quadrats.
(TIF)

Text S1 **The Information Theoretical approach: procedures for model selection with AICc and model averaging.**
(DOCX)

Table S1 **Median** **shelter volume per quadrat:** A) Predictors and interaction terms included in the four best models explaining variation in median shelter volume in 30 25-m^2^ quadrats located in three zones (RC =  reef crest, SG =  spur and groove, FS =  fragmented spurs) on two reefs (NB =  North Bellairs reef, CH =  Chefette reef). B) Predictors and interaction terms included in the five best models explaining variation in median shelter volume in 29 quadrats (after excluding the extreme median shelter volume of Quadrat 5). Zones and reefs were used as random nested factors in the models. Variables included in the different models are denoted by “•”. Predictors for which the 95% confidence interval (CI) did not overlap zero are indicated in bold. The number of parameters (K) used in each model, the AICc, the ΔAICc (AIC of model*_i_*−AIC of best model), the w*_im_* (normalized Akaike weights for each candidate model) and the deviance explained are shown at the bottom of the table. Model averaged estimates of parameters (β), unconditional standard errors (SE), 95% CI and the normalized Akaike weight for each predictor (w*_ip_*) are also shown. All models include a constant.
(DOCX)

Table S2 **Shelter occupancy:** A) Predictors included in the four best models explaining variation in shelter occupancy in 30 25-m^2^ quadrats located in three reef zones (RC =  reef crest, SG =  spur and groove, FS =  fragmented spurs) on two reefs (NB =  North Bellairs reef, CH =  Chefette reef), B) Predictors and interaction terms included in the best two models explaining variation in shelter occupancy in 29 quadrats (after excluding the extreme median shelter volume value found in Quadrat 5). Zones and reefs were used as random nested factors. Variables included in the different models are denoted by “•”. Predictors for which the 95% confidence interval (CI) did not overlap zero are indicated in bold. The number of parameters (K) used in each model, the AICc, the ΔAICc (AIC of model*_i_*−AIC of best model), the w*_im_* (normalized Akaike weights for each candidate model) and the deviance explained are shown at the bottom of the table. Model averaged estimates of parameters (β), unconditional standard errors (SE), 95% CI and the normalized Akaike weight for each predictor (w*_ip_*) are also shown. All models include a constant.
(DOCX)

Table S3 **Fish abundance and diversity in holes and overhangs.** Abundance of fishes found in shelters (holes and overhangs) in 30 quadrats sampled on two fringing reefs in Barbados.
(DOCX)
