# Supplementary figures and images for: Shelters and Their Use by Fishes on Fringing Coral Reefs
Source: PLoS One. 2012 Jun 20;7(6):e38450. doi: 10.1371/journal.pone.0038450 (PMC3380059; doi:10.1371/journal.pone.0038450)

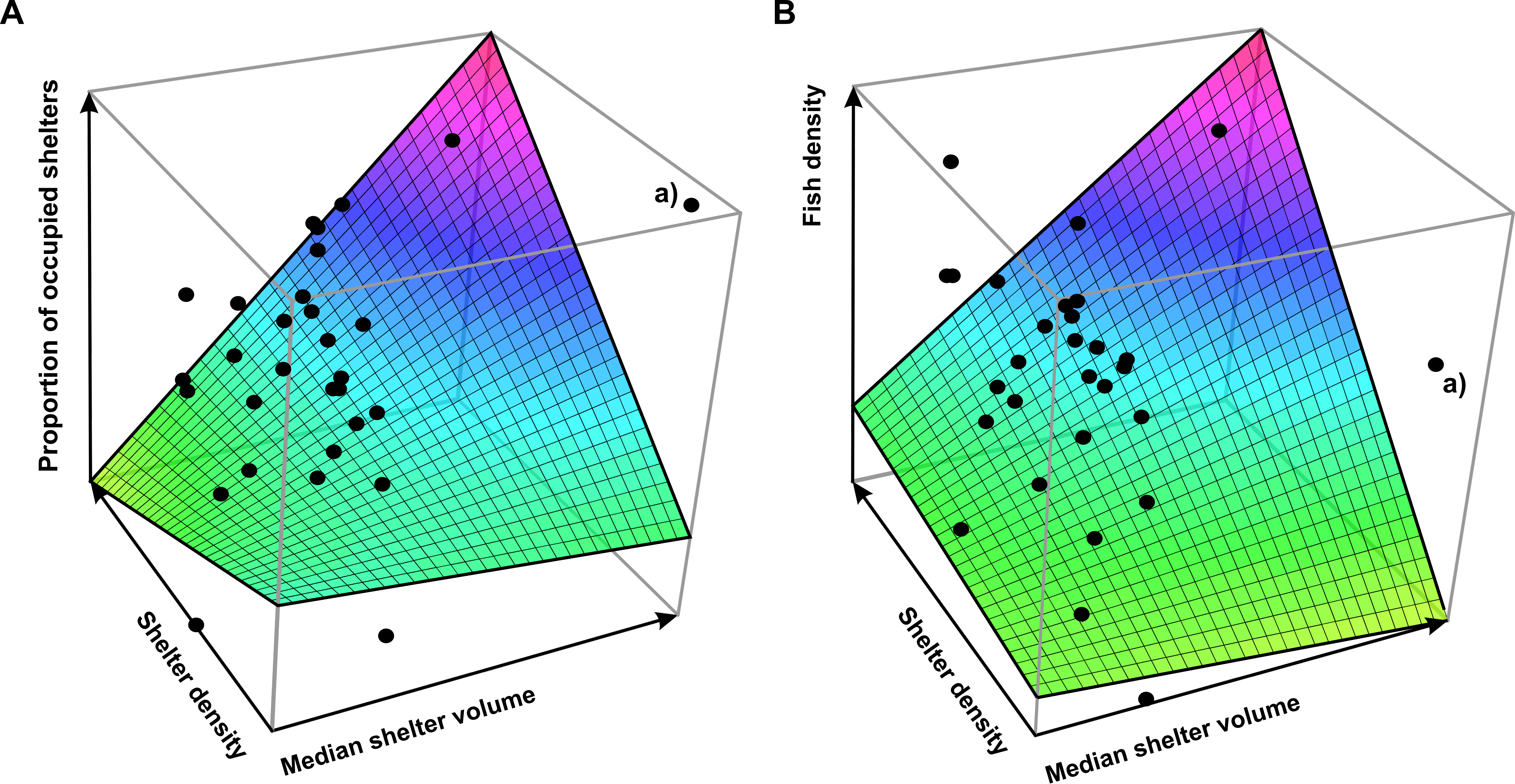

Supplement: Figure S1 — Shelter occupancy and fish density in relation to shelter density and median shelter volume. Three-dimensional plots showing A) the proportion of shelters occupied and B) fish density as a function of shelter density (y-axis) and median shelter volume (x-axis) for the 30 quadrats sampled. Black dots represent individual quadrats and the relationships shown by the colored grid were extracted from a general linear model. Median shelter volume was log10 transformed and all variables were z-standardized. Point a) represents the extreme value of median shelter volume in Quadrat 5, which is discussed in the text. (TIF) [file pone.0038450.s001.tif]

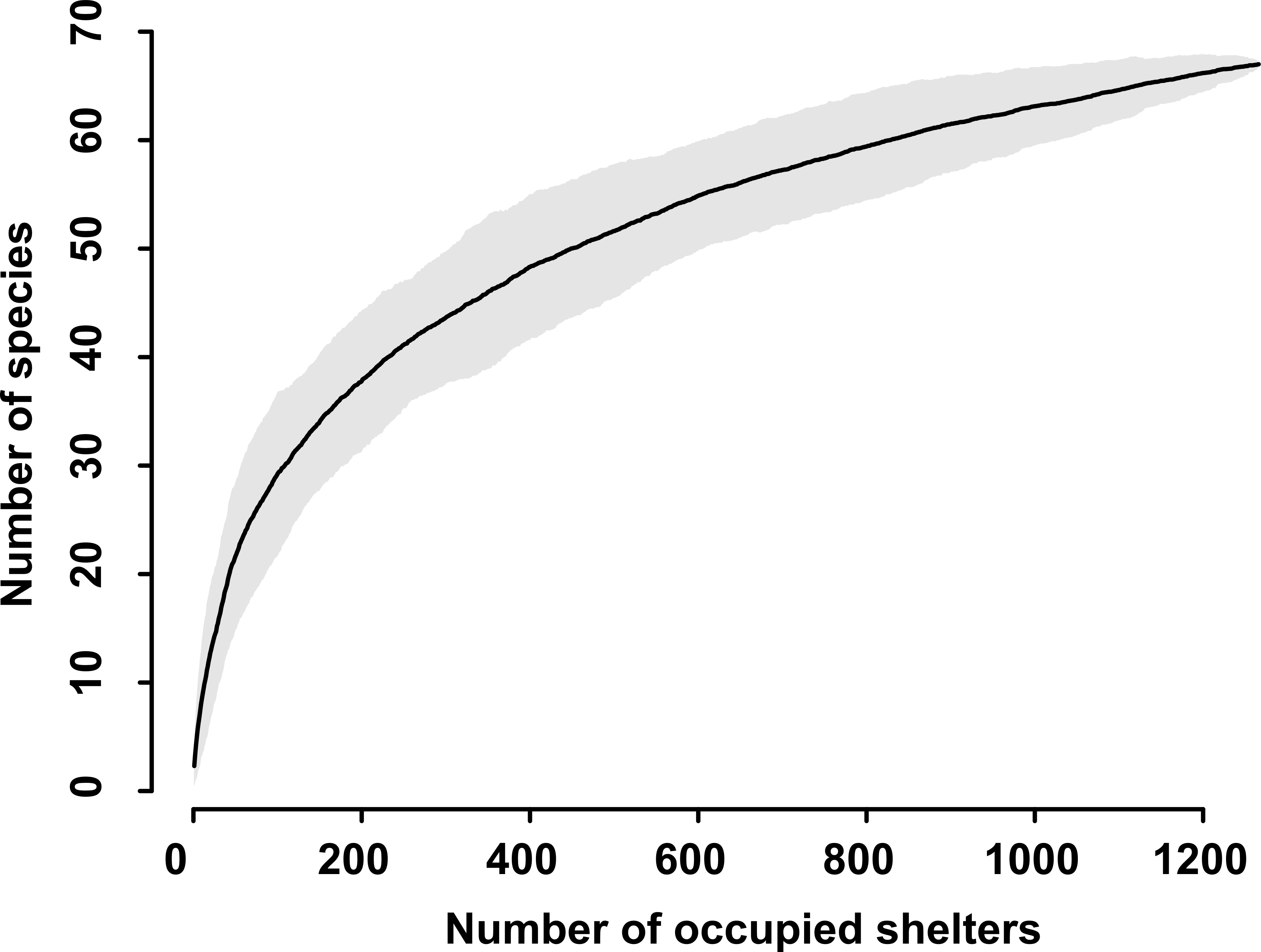

Supplement: Figure S2 — Rarefaction curve in shelter-using fish. Shelter-based rarefaction curve (solid line) ± standard deviation (shaded area) relating the expected number of species observed to the number of occupied shelters sampled across all 30 quadrats. (TIF) [file pone.0038450.s002.tif]
