# Supplementary material for: Shelters and Their Use by Fishes on Fringing Coral Reefs
Source: PLoS One. 2012 Jun 20;7(6):e38450. doi: 10.1371/journal.pone.0038450 (PMC3380059; doi:10.1371/journal.pone.0038450)
Supplement: Text S1 — The Information Theoretical approach: procedures for model selection with AICc and model averaging. (DOCX) [file pone.0038450.s003.docx]

**Text S1. The Information Theoretical approach: procedures for model selection with AICc and model averaging**

The AICc value for each model quantifies its parsimony (based on the trade-off between the model fit and the number of parameters included) relative to the other models considered. Candidate models are ranked using ΔAICc values (ΔAICc = AICc*_i_* − AICc_min_; where AICc_min_ represents the best model in the model subset). The plausibility of each model is quantified by its relative likelihood (*L*(model|data)), which is proportional to the exponent of −0.5×ΔAICc given the data. Then, for each candidate model, the normalized Akaike weights (*w_im_*; exp -0:5 x ΔAICc*_i_*) / exp -0.5 x ΔAICc*_r_*) are compiled as evidence that model *i* is the best of a set [32]. As a general rule of thumb, the confidence set of candidate models includes all models for which *w_im_* falls within 10% of the maximum normalized weight, suggesting that these models have substantial support in explaining the data [32]. Model averaging provides unconditional model variances and more reliable parameter estimates for each predictor. To determine the reliability of the predictor estimates from averaging, we calculated the weighted unconditional standard error with its associated confidence intervals (95% CI). We then used the normalized Akaike weights to assess the relative importance of each predictor (*w_ip_*) by summing the Akaike weights (*w_im_*) calculated for each model that contained the parameter of interest. Important predictors are included in either some or all of the best-fitting models and their normalized Akaike weight (w*_ip_*) approaches 1.0 while their 95% CI does not overlap zero.
