# Supplementary material for: Shelters and Their Use by Fishes on Fringing Coral Reefs
Source: PLoS One. 2012 Jun 20;7(6):e38450. doi: 10.1371/journal.pone.0038450 (PMC3380059; doi:10.1371/journal.pone.0038450)
Supplement: Table S1 — Median shelter volume per quadrat: A) Predictors and interaction terms included in the four best models explaining variation in median shelter volume in 30 25-m2 quadrats located in three zones (RC = reef crest, SG = spur and groove, FS = fragmented spurs) on two reefs (NB = North Bellairs reef, CH = Chefette reef). B) Predictors and interaction terms included in the five best models explaining variation in median shelter volume in 29 quadrats (after excluding the extreme median shelter volume of Quadrat 5). Zones and reefs were used as random nested factors in the models. Variables included in the different models are denoted by “•”. Predictors for which the 95% confidence interval (CI) did not overlap zero are indicated in bold. The number of parameters (K) used in each model, the AICc, the ΔAICc (AIC of modeli−AIC of best model), the wim (normalized Akaike weights for each candidate model) and the deviance explained are shown at the bottom of the table. Model averaged estimates of parameters (β), unconditional standard errors (SE), 95% CI and the normalized Akaike weight for each predictor (wip) are also shown. All models include a constant. (DOCX) [file pone.0038450.s004.docx]

**Table S1.** **Median** **shelter volume per quadrat**: A) Predictors and interaction terms included in the four best models explaining variation in median shelter volume in 30 25-m^2^ quadrats located in three zones (RC = reef crest, SG = spur and groove, FS = fragmented spurs) on two reefs (NB = North Bellairs reef, CH = Chefette reef). B) Predictors and interaction terms included in the five best models explaining variation in median shelter volume in 29 quadrats (after excluding the extreme median shelter volume of Quadrat 5). Zones and reefs were used as random nested factors in the models. Variables included in the different models are denoted by “⚫”. Predictors for which the 95% confidence interval (CI) did not overlap zero are indicated in bold. The number of parameters (K) used in each model, the AICc, the ΔAICc (AIC of model*_i_*−AIC of best model), the w*_im_* (normalized Akaike weights for each candidate model) and the deviance explained are shown at the bottom of the table. Model averaged estimates of parameters (β), unconditional standard errors (SE), 95% CI and the normalized Akaike weight for each predictor (w*_ip_*) are also shown. All models include a constant.

A) Models including Quadrat 5

| **Predictors** | **Model Rank** | | | | **β** | **SE** | **95% CI** | **w*_ip_*** |
| --- | --- | --- | --- | --- | --- | --- | --- | --- |
|  | 1 | 2 | 3 | 4 |  |  |  |  |
| *Constant* | ⚫ | ⚫ | ⚫ | ⚫ | 0.705 | 0.614 | -0.498 to 1.909 | 1.000 |
| **Rugosity** | ⚫ | ⚫ | ⚫ | ⚫ | **0.582** | **0.281** | **0.032 to 1.132** | **1.000** |
| Zone RC vs. FS | ⚫ | ⚫ |  | ⚫ | -0.492 | 0.415 | -1.306 to 0.321 | 0.795 |
| **Zone SG vs. FS** | ⚫ | ⚫ |  | ⚫ | **-1.226** | **0.508** | **-2.222 to -0.230** | **0.795** |
| Site | ⚫ |  |  | ⚫ | -0.275 | 0.202 | -0.672 to 0.121 | 0.492 |
| Rugosity * Zone RC vs. FS |  |  |  | ⚫ | -0.049 | 0.112 | -0.269 to 0.172 | 0.165 |
| Rugosity * Zone SG vs. FS |  |  |  | ⚫ | -0.126 | 0.150 | -0.421 to 0.168 | 0.165 |
| No. of parameters (K) | 6 | 5 | 3 | 8 |  |  |  |  |
| AICc | 84.71 | 84.86 | 85.64 | 86.07 |  |  |  |  |
| Δ AICc | 0.000 | 0.148 | 0.931 | 1.359 |  |  |  |  |
| w*_im_* | 0.326 | 0.303 | 0.205 | 0.165 |  |  |  |  |
| Deviance explained | 39.5 | 31.7 | 10.2 | 37.8 |  |  |  |  |

B) Models after excluding Quadrat 5

| **Predictors** | **Model Rank** | | | | | **β** | **SE** | **95% CI** | **w*_ip_*** |
| --- | --- | --- | --- | --- | --- | --- | --- | --- | --- |
|  | 1 | 2 | 3 | 4 | 5 |  |  |  |  |
| *Constant* | ⚫ | ⚫ | ⚫ | ⚫ | ⚫ | 0.045 | 0.305 | -0.553 to 0.642 | 1.000 |
| Rugosity |  | ⚫ | ⚫ |  | ⚫ | 0.082 | 0.084 | -0.082 to 0.247 | 0.496 |
| Zone RC vs. FS | ⚫ | ⚫ |  |  | ⚫ | -0.087 | 0.211 | -0.501 to 0.328 | 0.639 |
| Zone SG vs. FS | ⚫ | ⚫ |  |  | ⚫ | -0.515 | 0.304 | -1.111 to 0.082 | 0.639 |
| Site |  |  |  | ⚫ | ⚫ | -0.039 | 0.080 | -0.196 to 0.118 | 0.251 |
| No. of parameters (K) | 4 | 5 | 3 | 3 | 6 |  |  |  |  |
| AICc | 63.16 | 64.13 | 64.26 | 64.64 | 65.87 |  |  |  |  |
| Δ AICc | 0.000 | 0.962 | 1.093 | 1.473 | 2.711 |  |  |  |  |
| w*_im_* | 0.341 | 0.211 | 0.197 | 0.163 | 0.088 |  |  |  |  |
| Deviance explained | 16.9 | 22.3 | 0.35 | 2.65 | 25.6 |  |  |  |  |
