# Supplementary material for: Shelters and Their Use by Fishes on Fringing Coral Reefs
Source: PLoS One. 2012 Jun 20;7(6):e38450. doi: 10.1371/journal.pone.0038450 (PMC3380059; doi:10.1371/journal.pone.0038450)
Supplement: Table S2 — Shelter occupancy: A) Predictors included in the four best models explaining variation in shelter occupancy in 30 25-m2 quadrats located in three reef zones (RC = reef crest, SG = spur and groove, FS = fragmented spurs) on two reefs (NB = North Bellairs reef, CH = Chefette reef), B) Predictors and interaction terms included in the best two models explaining variation in shelter occupancy in 29 quadrats (after excluding the extreme median shelter volume value found in Quadrat 5). Zones and reefs were used as random nested factors. Variables included in the different models are denoted by “•”. Predictors for which the 95% confidence interval (CI) did not overlap zero are indicated in bold. The number of parameters (K) used in each model, the AICc, the ΔAICc (AIC of modeli−AIC of best model), the wim (normalized Akaike weights for each candidate model) and the deviance explained are shown at the bottom of the table. Model averaged estimates of parameters (β), unconditional standard errors (SE), 95% CI and the normalized Akaike weight for each predictor (wip) are also shown. All models include a constant. (DOCX) [file pone.0038450.s005.docx]

**Table S2.** **Shelter occupancy**: A) Predictors included in the four best models explaining variation in shelter occupancy in 30 25-m^2^ quadrats located in three reef zones (RC = reef crest, SG = spur and groove, FS = fragmented spurs) on two reefs (NB = North Bellairs reef, CH = Chefette reef), B) Predictors and interaction terms included in the best two models explaining variation in shelter occupancy in 29 quadrats (after excluding the extreme median shelter volume value found in Quadrat 5). Zones and reefs were used as random nested factors. Variables included in the different models are denoted by “⚫”. Predictors for which the 95% confidence interval (CI) did not overlap zero are indicated in bold. The number of parameters (K) used in each model, the AICc, the ΔAICc (AIC of model*_i_*−AIC of best model), the w*_im_* (normalized Akaike weights for each candidate model) and the deviance explained are shown at the bottom of the table. Model averaged estimates of parameters (β), unconditional standard errors (SE), 95% CI and the normalized Akaike weight for each predictor (w*_ip_*) are also shown. All models include a constant.

A) Models including Quadrat 5

| **Predictors** | **Model Rank** | | | | **β** | **SE** | **95% CI** | **w*_ip_*** |
| --- | --- | --- | --- | --- | --- | --- | --- | --- |
|  | 1 | 2 | 3 | 4 |  |  |  |  |
| *Constant* | ⚫ | ⚫ | ⚫ | ⚫ | -0.540 | 0.650 | -1.813 to 0.734 | 1.000 |
| **Median shelter volume** | ⚫ | ⚫ | ⚫ | ⚫ | **0.905** | **0.242** | **0.430 to 1.380** | 1.000 |
| Shelter density | ⚫ | ⚫ | ⚫ |  | -0.151 | 0.186 | -0.516 to 0.214 | 0.899 |
| Zone RC vs. FS |  | ⚫ | ⚫ |  | 0.613 | 0.372 | -0.116 to 1.341 | 0.531 |
| Zone SG vs. FS |  | ⚫ | ⚫ |  | 0.911 | 0.557 | -0.179 to 2.002 | 0.531 |
| Reefs NB vs. CH |  |  | ⚫ |  | 0.135 | 0.123 | -0.105 to 0.376 | 0.213 |
| **Shelter volume * shelter density** | ⚫ | ⚫ | ⚫ |  | **0.496** | **0.191** | **0.121 to 0.871** | **0.899** |
| No. of parameters (K) | 5 | 7 | 8 | 3 |  |  |  |  |
| AICc | 80.30 | 80.59 | 81.39 | 82.88 |  |  |  |  |
| Δ AICc | 0.000 | 0.292 | 1.091 | 2.583 |  |  |  |  |
| w*_im_* | 0.368 | 0.318 | 0.213 | 0.101 |  |  |  |  |
| Deviance explained | 30.8 | 43.7 | 51.7 | 21.3 |  |  |  |  |

B) Models after excluding Quadrat 5

| **Predictors** | **Model Rank** | | **β** | **SE** | **95% CI** | **w*_ip_*** |
| --- | --- | --- | --- | --- | --- | --- |
|  | 1 | 2 |  |  |  |  |
| *Constant* | ⚫ | ⚫ | -0.215 | 0.410 | -1.017 to 0.588 | 1.000 |
| Median shelter volume (Msv) |  | ⚫ | 0.143 | 0.126 | -0.105 to 0.390 | 0.139 |
| Total shelter volume | ⚫ |  | 0.206 | 0.156 | -0.100 to 0.513 | 0.861 |
| Shelter density |  | ⚫ | -0.045 | 0.046 | -0.135 to 0.046 | 0.139 |
| Zone RC vs. FS |  | ⚫ | 0.176 | 0.161 | -0.139 to 0.492 | 0.139 |
| Zone SG vs. FS |  | ⚫ | 0.265 | 0.245 | -0.215 to 0.744 | 0.139 |
| Reefs NB vs. CH |  | ⚫ | 0.088 | 0.085 | -0.080 to 0.255 | 0.139 |
| Msv * shelter density |  | ⚫ | 0.094 | 0.085 | -0.073 to 0.260 | 0.139 |
| No. of parameters (K) | 3 | 8 |  |  |  |  |
| AICc | 69.50 | 73.15 |  |  |  |  |
| Δ AICc | 0.000 | 3.653 |  |  |  |  |
| w*_im_* | 0.861 | 0.139 |  |  |  |  |
| Deviance explained | 4.33 | 59.2 |  |  |  |  |
