# Supplementary material for: Shelters and Their Use by Fishes on Fringing Coral Reefs
Source: PLoS One. 2012 Jun 20;7(6):e38450. doi: 10.1371/journal.pone.0038450 (PMC3380059; doi:10.1371/journal.pone.0038450)
Supplement: Table S3 — Fish abundance and diversity in holes and overhangs. Abundance of fishes found in shelters (holes and overhangs) in 30 quadrats sampled on two fringing reefs in Barbados. (DOCX) [file pone.0038450.s006.docx]

**Table S3.** **Fish abundance and diversity in holes and overhangs**. Abundance of fishes found in shelters (holes and overhangs) in 30 quadrats sampled on two fringing reefs in Barbados.

| **Family** | **Species** | **Common name** | **Holes** | **Overhangs** | **Total** |
| --- | --- | --- | --- | --- | --- |
|  |  |  | **(n=1 287)** | **(n= 558)** | **(n = 1 845)** |
| Acanthuridae | *Acanthurus coeruleus* | Blue tang | 1 | 2 | 3 |
| Apogonidae | *Apogon affinis* | Bigtooth cardinalfish | 1 | 0 | 1 |
| Apogonidae | *Apogon aurolineatus* | Bridle cardinalfish | 3 | 0 | 3 |
| Apogonidae | *Apogon binotatus* | Barred cardinalfish | 58 | 28 | 86 |
| Apogonidae | *Apogon evermanni* | Oddscale cardinalfish | 25 | 6 | 31 |
| Apogonidae | *Apogon maculatus* | Flamefish | 33 | 13 | 46 |
| Apogonidae | *Apogon phenax* | Mimic cardinalfish | 4 | 0 | 4 |
| Apogonidae | *Apogon pseudomaculatus* | Twospot cardinalfish | 3 | 0 | 3 |
| Apogonidae | *Apogon quadrisquamatus* | Sawcheek cardinalfish | 21 | 4 | 25 |
| Apogonidae | *Phaeoptyx pigmentaria* | Dusky cardinalfish | 13 | 0 | 13 |
| Blenniidae | *Ophioblennius macclurei* | Redlip blenny | 13 | 7 | 20 |
| Blenniidae | ? | Blenny unknown | 2 | 1 | 3 |
| Chaetodontidae | *Chaetodon striatus* | Banded butterflyfish | 1 | 0 | 1 |
| Cirrhitidae | *Amblycirrhitus pinos* | Redspotted hawkfish | 10 | 5 | 15 |
| Gobiidae | *Coryphopterus glaucofraenum* | Pallid goby/bridled goby | 2 | 4 | 6 |
| Gobiidae | *Elacatinus prochilos* and *E. evelynae* | Cleaner goby | 34 | 24 | 58 |
| Gobiidae | *Gnatholepis thompsoni* | Goldspot goby | 7 | 2 | 9 |
| Gobiidae | *Priolepis hipoliti* | Rusty goby | 2 | 0 | 2 |
| Grammatidae | *Gramma loreto* | Fairy basslet | 4 | 5 | 9 |
| Haemulidae | *Haemulon chrysargyreum* | Smallmouth grunt | 7 | 11 | 18 |
| Haemulidae | *Haemulon flavolineatum* | French grunt | 14 | 24 | 38 |
| Haemulidae | *Haemulon striatum* | Striped grunt | 1 | 0 | 1 |
| Holocentridae | *Sargocentron vexillarium* | Dusky squirrelfish | 109 | 22 | 131 |
| Holocentridae | *Neoniphon marianus* | Longjaw squirrelfish | 5 | 1 | 6 |
| Holocentridae | *Holocentrus rufus* | Longspine squirrelfish | 31 | 16 | 47 |
| Holocentridae | *Myripristis jacobus* | Blackbar soldierfish | 56 | 16 | 72 |
| Holocentridae | *Plectrypops retrospinis* | Cardinal soldierfish | 2 | 0 | 2 |
| Labridae | *Bodianus rufus* | Spanish hogfish | 2 | 2 | 4 |
| Labridae | *Halichoeres garnoti* | Yellowhead wrasse | 0 | 1 | 1 |
| Labridae | *Halichoeres maculipinna* | Clown wrasse | 6 | 0 | 6 |
| Labridae | *Halichoeres radiatus* | Puddingwife | 0 | 12 | 12 |
| Labridae | *Thalassoma bifasciatum* | Bluehead wrasse | 0 | 5 | 5 |

**Table S3.** Continued and concluded

| Labrisomidae | *Labrisomus bucciferus* | Puffcheek blenny | 16 | 5 | 21 |
| --- | --- | --- | --- | --- | --- |
| Labrisomidae | *Labrisomus kalisherae* | Downy blenny | 4 | 0 | 4 |
| Labrisomidae | *Labrisomus nuchipinnis* | Hairy blenny | 0 | 2 | 2 |
| Labrisomidae | *Malacoctenus aurolineatus* | Goldline blenny | 0 | 1 | 1 |
| Labrisomidae | *Malacoctenus triangulatus* | Saddled blenny | 12 | 11 | 23 |
| Labrisomidae | *Malacoctenus macropus* | Rosy blenny | 2 | 1 | 3 |
| Lutjanidae | *Lutjanus mahogoni* | Mahogany snapper | 0 | 3 | 3 |
| Monacanthidae | *Cantherhines pullus* | Orangespot filefish | 2 | 0 | 2 |
| Muraenidae | *Enchelycore carychroa* | Chestnut moray | 1 | 1 | 2 |
| Muraenidae | *Echidna catenata* | Chain moray | 3 | 0 | 3 |
| Muraenidae | *Gymnothorax miliaris* | Goldentail moray | 5 | 0 | 5 |
| Muraenidae | *Gymnothorax moringa* | Spotted moray | 6 | 1 | 7 |
| Ostraciidae | *Lactophrys triqueter* | Smooth trunkfish | 1 | 0 | 1 |
| Pempheridae | *Pempheris schomburgki* | Glassy sweeper | 2 | 2 | 4 |
| Pomacanthidae | *Pomacanthus paru* | French angelfish | 1 | 0 | 1 |
| Pomacentridae | *Abudefduf saxatilis* | Sergeant major | 0 | 1 | 1 |
| Pomacentridae | *Chromis multilineata* | Brown chromis | 26 | 30 | 56 |
| Pomacentridae | *Microspathodon chrysurus* | Yellowtail damselfish | 23 | 3 | 26 |
| Pomacentridae | *Stegastes adustus* | Dusky damselfish | 223 | 77 | 300 |
| Pomacentridae | *Stegastes diencaeus* | Longfin damselfish | 320 | 152 | 472 |
| Pomacentridae | *Stegastes partitus* | Bicolor damselfish | 80 | 25 | 105 |
| Pomacentridae | *Stegastes planifrons* | Threespot damselfish | 31 | 9 | 40 |
| Pomacentridae | *Stegastes variabilis* | Cocoa damselfish | 3 | 1 | 4 |
| Priacanthidae | *Heteropriacanthus cruentatus* | Glasseye snapper | 0 | 1 | 1 |
| Scaridae | *Scarus vetula* | Queen parrotfish | 1 | 0 | 1 |
| Scaridae | *Sparisoma viride* | Stoplight parrotfish | 2 | 4 | 6 |
| Sciaenidae | *Equetus punctatus* | Spotted drum | 2 | 0 | 2 |
| Scorpaenidae | *Scorpaena plumieri* | Spotted scorpionfish | 1 | 1 | 2 |
| Serranidae | *Cephalopholis cruentatus* | Graysby | 7 | 4 | 11 |
| Serranidae | *Epinephelus adscensionis* | Rock hind | 1 | 0 | 1 |
| Serranidae | *Rypticus saponaceus* | Greater soapfish | 30 | 8 | 38 |
| Serranidae | *Rypticus subbifrenatus* | Spotted soapfish | 4 | 0 | 4 |
| Syngnathidae | *Cosmocampus elucens* | Shortfin pipefish | 2 | 0 | 2 |
| Tetraodontidae | *Canthigaster rostrata* | Sharpnose puffer | 6 | 4 | 10 |
